# Isoprenaline/β2-AR activates Plexin-A1/VEGFR2 signals via VEGF secretion in gastric cancer cells to promote tumor angiogenesis

**DOI:** 10.1186/s12885-017-3894-0

**Published:** 2017-12-20

**Authors:** Yanjie Lu, Qian Xu, Yanzhen Zuo, Lei Liu, Shaochen Liu, Lei Chen, Kang Wang, Yuntao Lei, Xiangyang Zhao, Yuhong Li

**Affiliations:** 1 Department of Pathology; Cancer Research Laboratory, Chengde Medical College, Shangerdaohezi Avenue, Chengde, Hebei 067000 People’s Republic of China; 2 Institute of Basic Medical Sciences, Chengde Medical College, Chengde, 067000 People’s Republic of China; 3 Department of Pharmacology, Chengde Medical College, Chengde, 067000 People’s Republic of China; 4 Department of Pathogenic Microorganism, Chengde Medical College, Chengde, 067000 People’s Republic of China; 5 Department of Pathology, Chengde Medical College, Chengde, 067000 People’s Republic of China; 6 Department of General Surgery, the 266th Hospital of Chinese People’s Liberation Army, Puning Avenue, Chengde, Hebei 067000 People’s Republic of China

**Keywords:** Gastric cancer, Angiogenesis, Isoprenaline, Plexin-A1, VEGFR2

## Abstract

**Background:**

The role of stress signals in regulating gastric cancer initiation and progression is not quite clear. It is known that stress signals modulate multiple processes such as immune function, cell migration and angiogenesis. However, few studies have investigated the mechanisms of how stress signals contribute to gastric cancer angiogenesis.

**Methods:**

Here, we used β2-adrenergic receptor (β2-AR) agonist isoprenaline to imitate a stress signal and demonstrated the molecular mechanism underlying stress’s influence on tumor angiogenesis.

**Results:**

We found that isoprenaline stimulated vascular endothelial growth factor (VEGF) secretion in gastric cancer cells and plexin-A1 expression was induced by human recombinant VEGF165 in both gastric cancer cells and vascular endothelial cells. Furthermore, interfere with plexin-A1 expression in gastric cancer cells influence HUVEC tube formation, migration and tumor growth in vivo.

**Conclusions:**

These findings suggest that isoprenaline stimulate VGEF secretion and subsequently up-regulate the expression of plexin-A1 and VEGFR2 in gastric cancer cells, which form a positive impetus to promote tumor angiogenesis. This study reveals a novel molecular mechanism that a stress signal like isoprenaline may enhance angiogenesis via activating plexin-A1/VEGFR2 signaling pathway in gastric cancer, which may be a potential target in development of an anti-angiogenic therapy for gastric cancer.

## Background

Gastric cancer is the fifth most common cancer and the third leading cause of cancer mortality worldwide, with nearly 950,000 new cases and nearly 723,000 deaths estimated in 2012 [1]. Tumor angiogenesis is closely related to poor prognosis. Angiogenesis stands for formation of new blood vessels, which plays a pivotal role in tumor growth and metastasis. Neo-vascularization is a multi-step progress, which involves the interactions between tumor cells and stromal endothelial cells through several growth factors and their receptors as well as activation of the pro-angiogenic intracellular signaling pathways. Anti-angiogenic therapies, i.e., targeting vascular endothelial growth factor (VEGF) and its receptors, have been developed in recent years. Blocking angiogenesis is a validated effective therapeutic approach [2], which has been successfully exploited in treating several tumor types including glioblastoma [3], colorectal cancer [4], and ovarian cancer [5]. However, in gastric cancer, the therapeutic role of anti-angiogenic agents has not been determined, especially for patients with advanced diseases where the treatment options are limited. The fact that targeting VEGF family receptors closely related to inhibition of VEGF-mediated signaling, proliferation and migration of endothelial cells [6] and anti-tumor activity in animal models, has been proven [7]. Targeting VEGF receptor2 (VEGFR2) has been tested in the patients with advanced gastric cancer in a Phase III study [8, 9]. However, this second-line therapy has not consistently translated into a survival advantage over standard treatment in randomized clinical trials. For example, Ramucirumab, which targets VEGFR2, had a marginal improvement in overall survival but did not achieve the expected outcome [10]. Thus, new anti-angiogenic therapeutics must be explored.

Recent evidence suggests that there is a link between stress and certain cancers [11–13]. Stress, chronic depression, negative social support and other psychological factors might activate the hypothalamic–pituitary–adrenal (HPA) axis, thus causing complex physiological and neuroendocrine changes. After the activation of neurosensory signals, the adrenal gland, brain, and sympathetic nerve terminals release catecholamines, glucocorticoids and other stress hormones [14].Under the role of these hormones, multiple tumor cell processes can be modulated, which contribute to immune function, cell migration, invasion, and angiogenesis [14]. Several studies have shown that catecholamines activate β_2_-adrenergic receptors (β_2_-AR) to interfere with bio-behaviors of tumor cells [15–17]. However, there has been very little research on stress-induced angiogenesis in gastric cancer. The objective of the present study is to determine the mechanisms of how β2-AR signaling pathway contributes to angiogenesis in gastric cancer.

Plexin-A1 is one of the semaphoring family receptors that are single-pass membrane-bound semaphorins initially identified as axon guidance factors. In recent years, plexin-A1 was found to play critical roles in tumor biology such as cell survival and anchorage-independent growth [18], as well as angiogenesis [19]. Interestingly, plexin-A1 forms a complex with VEGFR2 when stimulated with sema6D during cardiac morphogenesis [20]. Previously, we found plexinA1 expression level had no correlation with the age of patients, tumor size, invasion depth, differentiation degree and lymph node metastasis. However, plexin-A1 was positively correlated with angiogenesis by detecting microvessel density (MVD) in gastric cancer [21]. Given that plexin-A1/VEGFR2 play a critical role in cardiac morphogenesis and angiogenesis [22], we further investigated the expression and functional relationship between plexin-A1 and VEGFR2 in gastric tumors and gastric cancer cell lines. Therefore, in the present study, we focused on the expression and functional relationship between plexin-A1 and VEGFR2 in the context of stress-activated signaling pathway.

## Methods

### Human tissue specimens:

Gastric tissues were obtained from the 266th hospital of People’s Liberation Army (PLA) and affiliated hospital of Chengde medical college with the institutional approval and informed consent of the patients. The procedures to obtain human gastric tissues were in accordance with the Ethical Principles for Medical Research Involving Human Subjects, as formulated in the World Medical Association Declaration of Helsinki (revised in 2008). During surgical resection, gastric tumor tissues and the normal gastric tissues approximately 5 cm away from the macroscopic margin of the resected tumors were obtained from 10 patients, who were diagnosed as having gastric adenocarcinoma by the pathologists. The patients’ ages were between 37 and 82 years old, with a median age of 58 years old. There were 6 male and 4 female patients. None of the patients had received any chemotherapy or radiotherapy prior to the surgery.

### Reagents

Roswell Park Memorial Institute (RPMI)-1640medium, fetal bovine serum (FBS), 0.25% trypsin and 0.02% EDTA were purchased from Gibco company (St. Louis, MO, USA); the M - MLV reverse transcription system and quantitative polymerase chain reaction (qPCR) master mix were purchased from Promega (Madison, WI, USA); anti-VEGFR2 mouse monoclonal antibody and anti-β2-AR rabbit monoclonal antibody, anti-plexin-A1 rabbit monoclonal antibody, anti-glyceraldehyde-3-phosphate dehydrogenase (GAPDH) mouse monoclonal antibody, anti-β-actin mouse monoclonal antibody and VEGF human ELISA kit were purchased from Abcam (Cambridge, MA, UK); peroxidase-conjugated affinipure goat anti-mouse IgG, peroxidase-conjugated affinipure goat anti-rabbit IgG, Alexa Fluor488-conjugated affinipure goat anti-mouse IgG and Alexa Fluor594-conjugated affinipure goat anti-rabbit IgG were purchased from Santa Cruz Biotechnology (Santa Cruz, CA, USA). Human recombinant VEGF165 was purchased from Protech Co., Ltd. (Rocky Hill, NJ, USA). ICI 118,551 hydrochloride (ICI), a selective antagonist of β2-AR, was purchased from Sigma-Aldrich (St. louis, MO, USA). High concentration Matrigel Basement Membrane Matirx was purchased from BD Biosciences (Bedford, MA, UK).

### Cell culture

Human gastric cancer cell lines poorly differentiated MGC803 [23]/undifferentiated HGC27 [24] and human umbilical vein endothelial cells (HUVECs) were provided by Chinese Academy of Military Medical Sciences. HUVEC, MGC803 and HGC27 were cultured in an incubator with an atmosphere of 5% CO_2_ at 37 °C in RPMI-1640 medium, supplemented with 10% FBS. The cells were then subcultured with 0.25% trypsinand 0.02% EDTA when the cells grew to approximately 90% confluent. The experiments were carried out using the cells growing at logarithmic growth phase.

### Quantitative real-time PCR analysis (qRT-PCR)

Total RNA was isolated using the RNAgents® Total RNA Isolation System (Promega) with DNase I (Invitrogen) treatment. 2 μg RNA, oligo (dt) 20 primers and the M-MLV first-strand synthesis system kit were used to synthesize cDNA. Real-time PCR was performed using the SYBR® Green I on GoTaq®qPCR Detection System (Promega) according to the manufacturer’s instructions. The results were analyzed using the comparative threshold cycle method with GAPDH as an internal control. Results were normalized to GAPDH levels using the formula ΔCt (Cycle threshold) = Ct of target gene – Ct of GAPDH. The mRNA level of the control group was used as the baseline; therefore, ΔΔCt was calculated using the formula ΔΔCt = ΔCt of target gene – ΔCt of the baseline. The fold change of mRNA level was calculated as fold = 2^-ΔΔCt^. Primers used in this study were synthesized by Hua Da Gene Technology (Shanghai, China) (see Table 1).

**Table 1:** Primers used in this study

Gene	Primer sequences
plexin-A1	5’-TGGACGACCTGTTTGAGACCA-3′ (Sense) 5’-TGATCACGTTCACCCAGAAGC-3′ (Antisense)
VEGFR2	5’-CTACCAGTACGGCACCACTCAA-3′ (Sense) 5’-TCTTCCTCCAACTGCCAATACC-3′ (Antisense)
GAPDH	5’-GAAGGTCGGAGTCAACGGAT-3’(Sense) 5’-CTGGAAGATGGTGATGGGATT-3′ (Antisense)

### Western blot analysis

Total proteins were extracted with RIPA buffer (Thermo Scientific, Rockford, IL, USA) from each group and quantified using a BCA kit (Thermo Scientific). The proteins were separated by sodium dodecyl sulfate-polyacrylamide gel electrophoresis (SDS-PAGE) and transferred to polyvinylidenedifluoride (PVDF) membranes. After blocking by 5% BSA, blots were probed with the appropriate primary antibodies overnight at 4 °C. The antibodies used include anti-β2-AR antibody (1:1000 dilution), anti-plexin-A1 antibody (1:1000 dilution), anti-VEGFR2 antibody (1:500 dilution), anti-β-actin antibody (1:1000dilution), and anti-GAPDH (1:500 dilution). The membranes were washed and incubated with horseradish peroxidase-conjugated secondary antibodies (goat anti-mouse or anti-rabbit; 1:1000dilution). Immunoreactive bands were detected using enhanced chemiluminescence (ECL) substrate (Pierce, Rockford, IL, USA) and imaged using the Image Quant LAS4000 system (GE Company, Pittsburgh, PA, USA).

### Immunohistochemistry and fluorescence microscopy

Immunohistochemical analysis was performed using standard techniques. Briefly, paraffin-embedded tissues were cut into 4-μm thick sections, deparaffinized, and antigen-recovered in citrate buffer. The sections were blocked for endogenous avidin, peroxidase and biotin, and then incubated with anti-plexin-A1 antibody, after washing 3 times, the staining was developed using the LSAB kit (DAKO, Glostrup, Denmark) according to the manufacturer’s instructions. For fluorescence immunohistochemical staining and microscopy, the sections were fixed in 4% paraformaldehyde for 30 min and then permeabilized in 0.2% Triton X-100 in phosphate-buffered saline for 10 min. The primary antibodies (mouse anti-VEGFR2 antibody, 1:100 dilution; rabbit anti-plexin-A1antibody, 1:100 dilution; rabbit anti-β2-AR antibody, 1:200dilution) were used in combination with appropriate Alexa-Fluor-conjugated secondary antibodies (1:200 dilution). The nuclei were stained using 4′, 6-diamidino-2-phenylindole (DAPI) (Vector Labs, H-1200, Southern Biotech, Birmingham, AL, USA). Fluorescence images were collected under a laser scanning confocal microscope (Leica, Solms, Germany).

### ELISA assay

We quantified concentrations of VEGF levels intracellular or extracellular of gastric cancer using a VEGF human ELISA kit. The tests were performed according to the manufacturer’s recommendations.

### Short hairpin RNA transfection

Short hairpin RNA (shRNA) plasmid vectors were purchased from Gene Pharma (Shanghai, China), including pGPU6/GFP/Neo-shPlexin-A1 (targeting plexin-A1) and pGPU6/GFP/Neo-shNC (control, without targeting any genes). The sequence of shPlexinA1 is as follows: CCGGGCACTTCTTCACGTCCAAGATCTCGAGATCTTGGACGTGAAGAAGTGCTTTTTG. The constructs were transfected into MGC803 cells with jetPRIME® (Polyplus Transfection, Strasbourg, France). The procedure of transfection was performed according to the manufacturer’s protocols. Then, stable cell clones were selected by treatment with 400-1000 μM antibiotics G418 (Solarbio Inc., Beijing, China) for 2 months. Antibiotics-resistant cell clones were verified for expression of green fluorescent protein (GFP).

### Endothelial cell transwell migration assay

We used the CORING (Tewksbury, MA, UK) transwell chamber (6.5 mm Diameter Inserts; 8-μm pore size; polycarbonate membrane) to quantify HUVEC migration. Briefly, the bottom chambers were seeded with MGC803 or HGC27 gastric cancer cells (1 ml/well) at a concentration of 4 × 10^5^ cells/ml. Then HUVEC (2 × 10^5^cells/ml) suspended in 200 μl completed medium and seeded onto the upper chambers. After being cultured at 37 °C for 24 h, the cells without penetrating the polycarbonate membrane were wiped off with cotton bud. The membrane was removed and fixed with 4% paraformaldehyde and stained with crystal violet solution. 5 fields were randomly selected under an Olympus microscope (Tokyo, Japan) and the number of cells was counted.

### Endothelial cell tube formation assay

BD Matrigel was pipette into prechilled 96-well plates (50 μl per well) and polymerized for at least 30 min. 50 μl HUVECs were seeded at 2 × 10^5^ cells/ml in serum-free medium plus 50 μl MGC803 or HGC27 cells at a concentration 2 × 10^5^ cells/ml. After incubation for 6 h, images of capillary-like structures were captured with an inverted microscope. The tube area was quantified by Image-Pro Plus 6.0 software.

### Animal husbandry and in vivo tumorigenicity assay

Animal study was performed at the Animal Experimental Center of the General Hospital of the People’s Liberation Army. Animal welfare and experimental procedures were carried out in accordance with the Guide for the Care and Use of Laboratory Animals (Ministry of Science and Technology of China, 2006), and were approved by the animal ethics committee of PLA. BALB/c nude mice (4–6 weeks old, half females and half males) were obtained from Charles River Laboratories Ltd., USA. The mice were kept in individually ventilated caging (IVC) systems with a standardized light/dark cycle, humidity of 40–70%, at 20–24 °C, and fed with a standard rodent laboratory diet irradiated with cobalt-60 and RO water ad libitum. The gastric cancer stably transfected with shPlexin-A1 or shNC were resuspended (1 × 10^8^cells/ml) in 100 μl of RPMI-1640 medium and injected subcutaneously into both sides of the posterior flanks of BALB/c nude mice. The blank control group was injected with 100 μl RPMI-1640 medium. 3 mice were used in each group and sacrificed after 3 weeks.

### Statistical analysis

All experiments were repeated at least 3 times. Data are presented as mean ± standard deviation (SD). Statistical analysis was performed using one-way analysis of variance (ANOVA) and Student’s *t* test. A *P*-value <0.05 was considered statistically significant and was indicated with asterisks in the figures.

## Results

### Plexin-A1 and VEGFR2 are highly expressed in both the tumor cells and the vascular endothelial cells within the gastric tumor

**Fig. 1:**
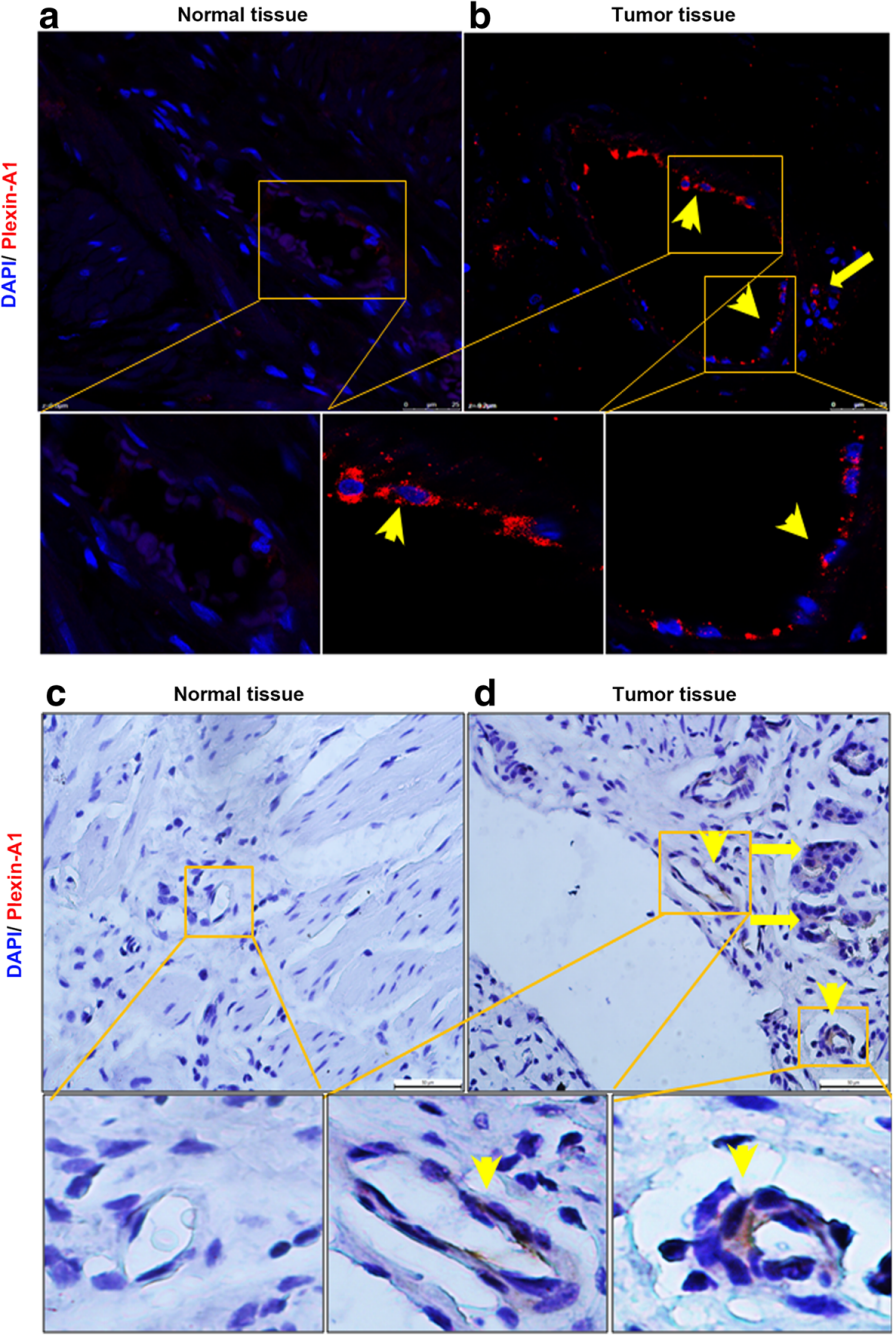
Plexin-A1 is highly expressed in both tumor cells and vascular endothelial cells. **a**-**b** Representative images of immunohistochemical fluorescent staining show that plexin-A1 was not expressed in normal gastric tissues (**a**), but highly expressed in gastric cancer cells (**b**, arrow) and vascular endothelial cells within the gastric cancer (**b**, arrowheads). The lower 3 panels are the magnified regions of the upper panels. Scale bar, 25 μm. **c**-**d** Representative images of immunohistochemical staining with DAB show that plexin-A1 was not expressed in normal gastric tissues (**c**), but highly expressed in gastric cancer **d**, arrow) and vascular endothelial cells within the gastric cancer (**d**, arrowheads). The lower 3 panels are the magnified regions of the upper panels. Scale bar, 50 μm

**Fig. 2:**
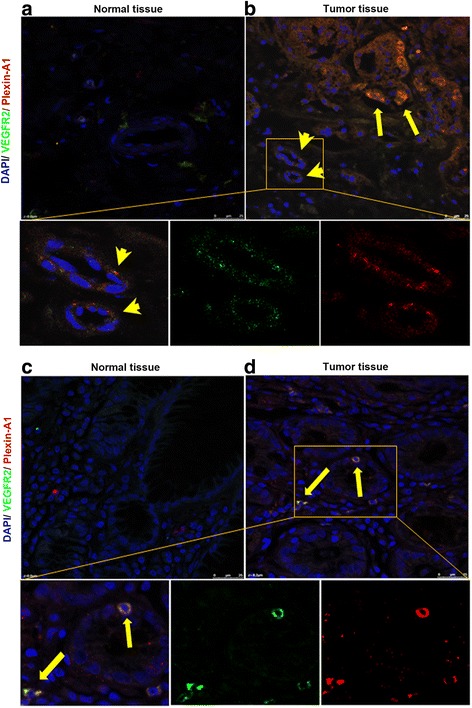
Plexin-A1 is co-localized with VEGFR2 in both gastric tumor cells and vascular endothelial cells. **a-b** Representative images of fluorescent double staining of plexin-A1 (in red color) and VEGFR2 (in green color) in the vascular endothelial cells within normal gastric tissues (**a**, no positive double staining) and within the gastric tumor (**b**, arrow indicates cancer cells that show positive co-localization of plexin-A1 and VEGFR2; arrowheads indicates vascular endothelial cells that show positive co-localization of plexin-A1 and VEGFR2). The lower 3 panels are the magnified region of panel. **b**: left, co-localization; middle, green color only; right, red color only. Scale bar, 25 μm. **c-d** Representative images of fluorescent double staining of plexin-A1 (in red color) and VEGFR2 (in green color) in the normal gastric tissues (**c**, no positive double staining) and the gastric tumor (**d**, arrow indicates cancer cells that show positive co-localization of plexin-A1 and VEGFR2). The lower 3 panels are the magnified region of panel: left, co-localization; middle, green color only; right, red color only. Scale bar, 25 μm

**Fig. 3:**
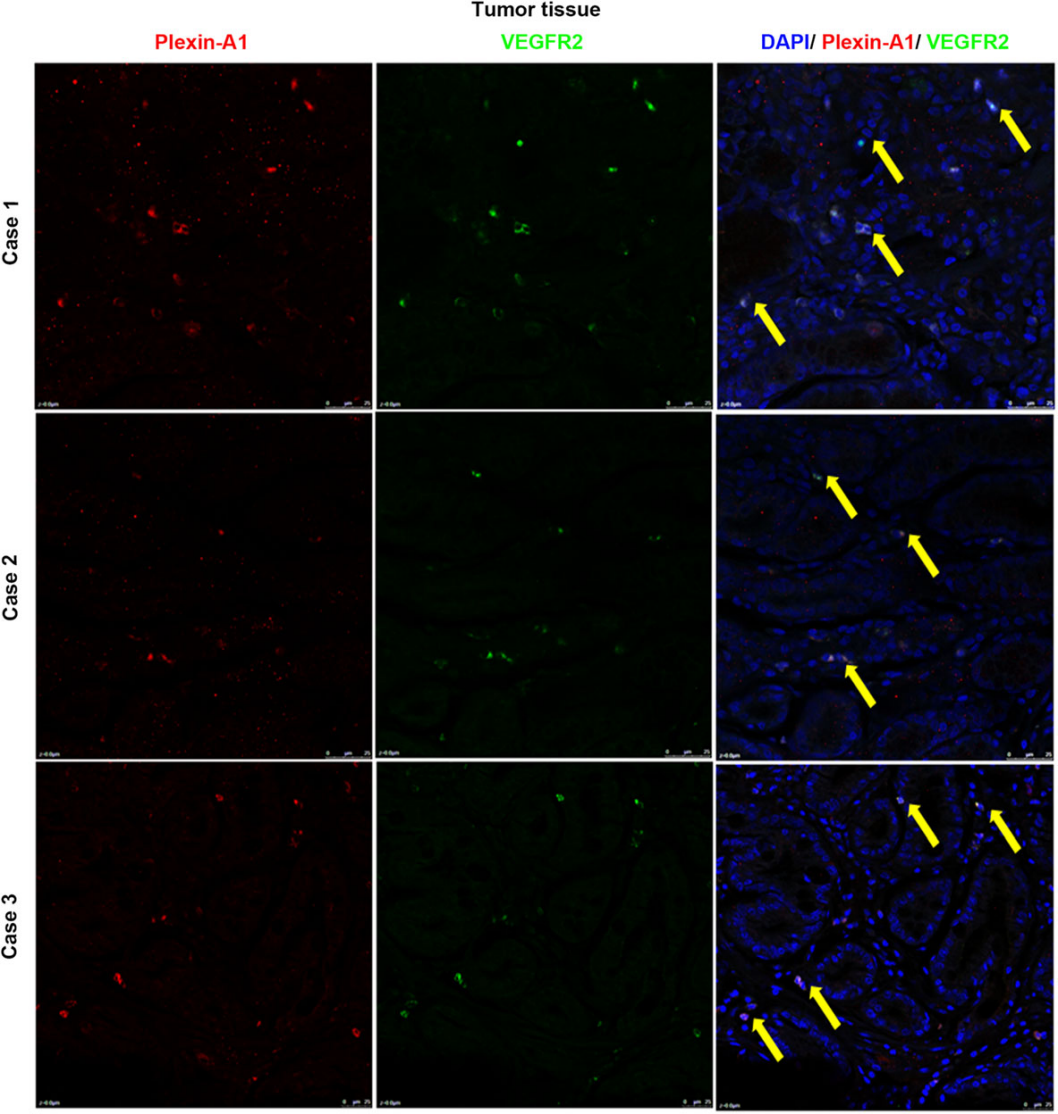
Plexin-A1 expression is co-localized with VEGFR2 in gastric cancer cells. Representative images of immunohistochemical fluorescent double staining of plexin-A1 (in red color, left column) and VEGFR2 (in green color, middle column) in gastric cancer; co-localization is shown in the right column (arrows). Scale bar, 25 μm

Using immunohistochemical and fluorescent staining, we found that plexin-A1 was highly expressed within gastric cancer, in both the tumor cells and the vascular endothelial cells within the tumor, but not in the adjacent normal gastric tissues (Fig. 1a-d). In addition, plexin-A1 and VEGFR2 signals appeared to localize simultaneously in the tumor-associated vascular endothelial cells and tumor cells, but not in the normal gastric tissues (Figs. 2a-d and 3). These findings indicated plexin-A1 and VEGFR2 may play a critical role in tumor angiogenesis.

### Isoprenaline promote tumor cells VEGF secretion, which can induce plexin-A1 expressed in tumor cells and HUVEC

**Fig. 4:**
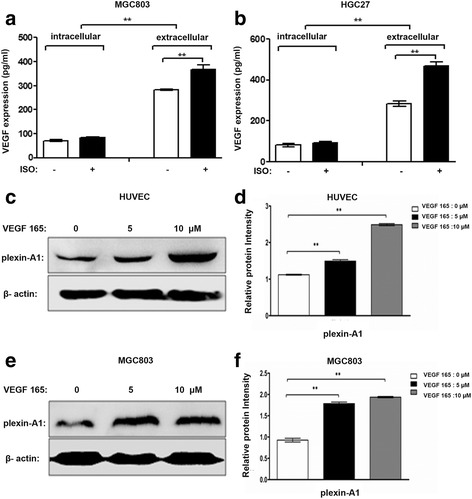
Isoprenaline promote tumor cells VEGF secretion and plexin-A1 is induced by VEGF165 in both HUVECs and gastric cancer cells. **a-b** MGC803 and HGC27 cells were treated with 5 μM ISO for 3 h after serum starvation; Elisa assay showed VEGF expression intracellular or extracellular of gastric cancer cells. **c**, **e** HUVECs and MGC803 cells were treated with 0, 5 or 10 μM of VEGF165 for 24 h after serum starvation; plexin-A1protein expression was analyzed by Western blot. The relative expression levels of plexin-A1 were shown in (**d**, **f**). Data represent mean ± SD (*n* = 3 for each group, *P < 0.05, **P < 0.01)

Human gastric cancer cell line MGC803 (Fig. 4a) and HGC27 (Fig. 4b) expressed significantly higher levels of VEGF after incubated with isoprenaline (ISO), which mainly secreted out of the cells. Human recombinant VEGF165 treatment up-regulated expression of plexin-A1 protein in a dose-dependent manner in both HUVECs (Fig. 4c-d) and MGC803 cells (Fig. 4e-f). These findings suggest that isoprenaline stimulate VEGF secretion in tumor cells, which further up-regulate plexin-A1 expression in HUVECs and gastric cancer cells.

### Isoprenaline promotes plexin-A1 and VEGFR2 expression via β2-AR in human gastric cancer cell lines

**Fig. 5:**
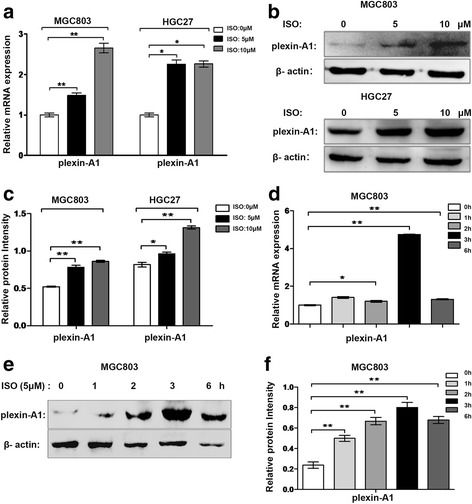
Isoprenaline promotes plexin-A1 expression in human gastric cancer cell lines. **a-c** MGC-803 and HGC-27 cells were treated with 0, 5 or 10 μM ISO for 3 h after serum starvation. **a** qRT-PCR analysis of plexin-A1 expression in MGC803 and HGC27 cells. **b-c** The expression of plexin-A1 protein was analyzed by Western blot and quantified. **d-f** MGC803 cells were starved overnight and treated with 5 μM ISO for 0, 1, 2, 3 and 6 h; the relative mRNA expression levels of plexin-A1 were analyzed using qRT-PCR (**d**) and the protein expression of plexin-A1 was analyzed by Western blot and quantified (**e-f**).Data represent mean ± SD (*n* = 3 for each group,*P < 0.05, **P < 0.01)

**Fig. 6:**
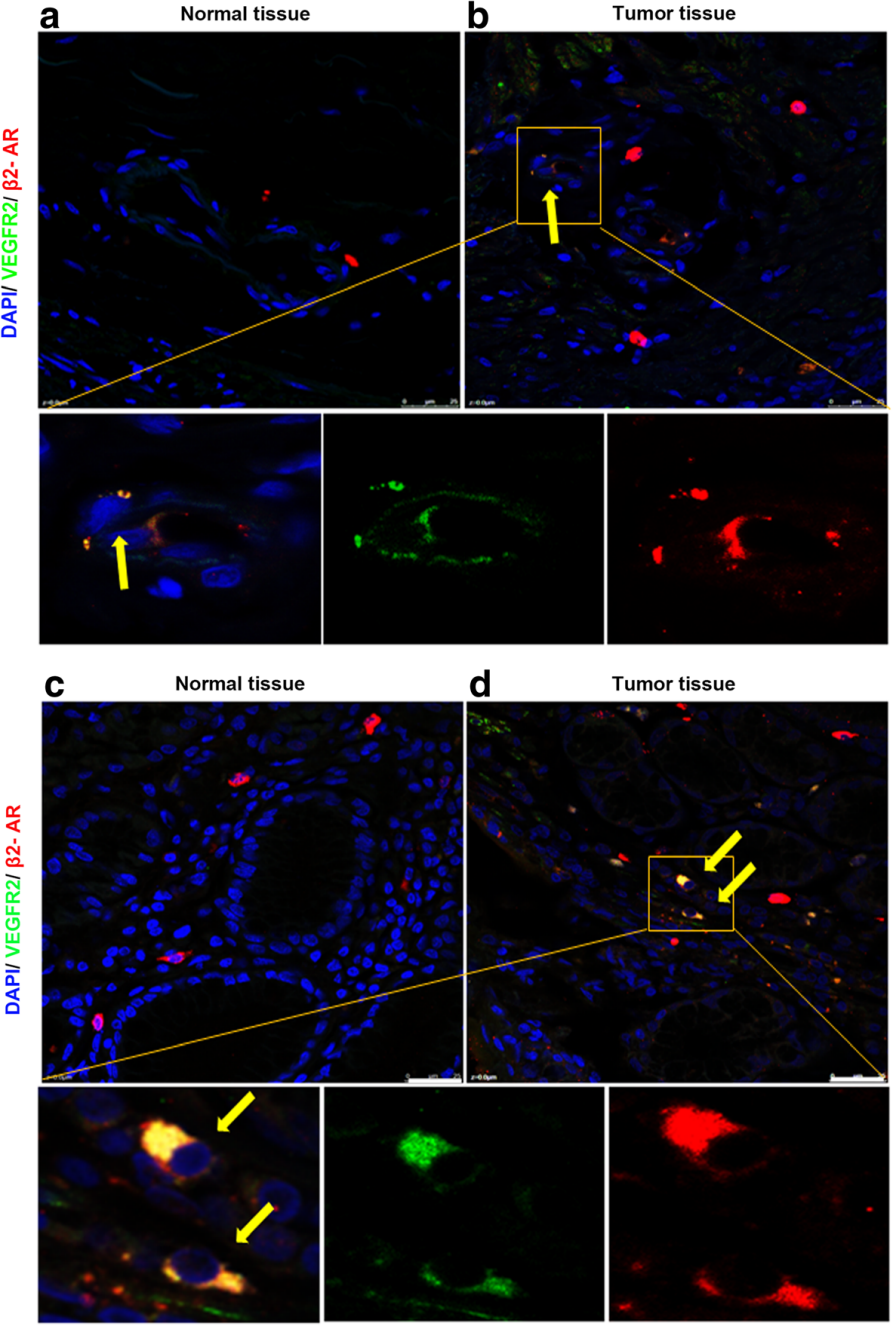
β2-AR and VEGFR2 are co-expressed in both vascular endothelial cells and gastric tumor cells. **a-d** Representative images of immunohistochemcal fluorescent staining show the double staining of β2-AR (in red color) and VEGFR2 (in green color) in the normal gastric tissues (**a** and **c,** no positive double staining), in the vascular endothelial cells within the gastric tumor (**b** and its 3 magnified panels: left, co-localization as indicated by an arrow; middle, green color only; and right, red color only), and in the gastric cancer cells (**d** and its 3 magnified panels: left, co-localization as indicated by an arrow; middle, green color only; and right, red color only). Scale bar, 25 μm

**Fig. 7:**
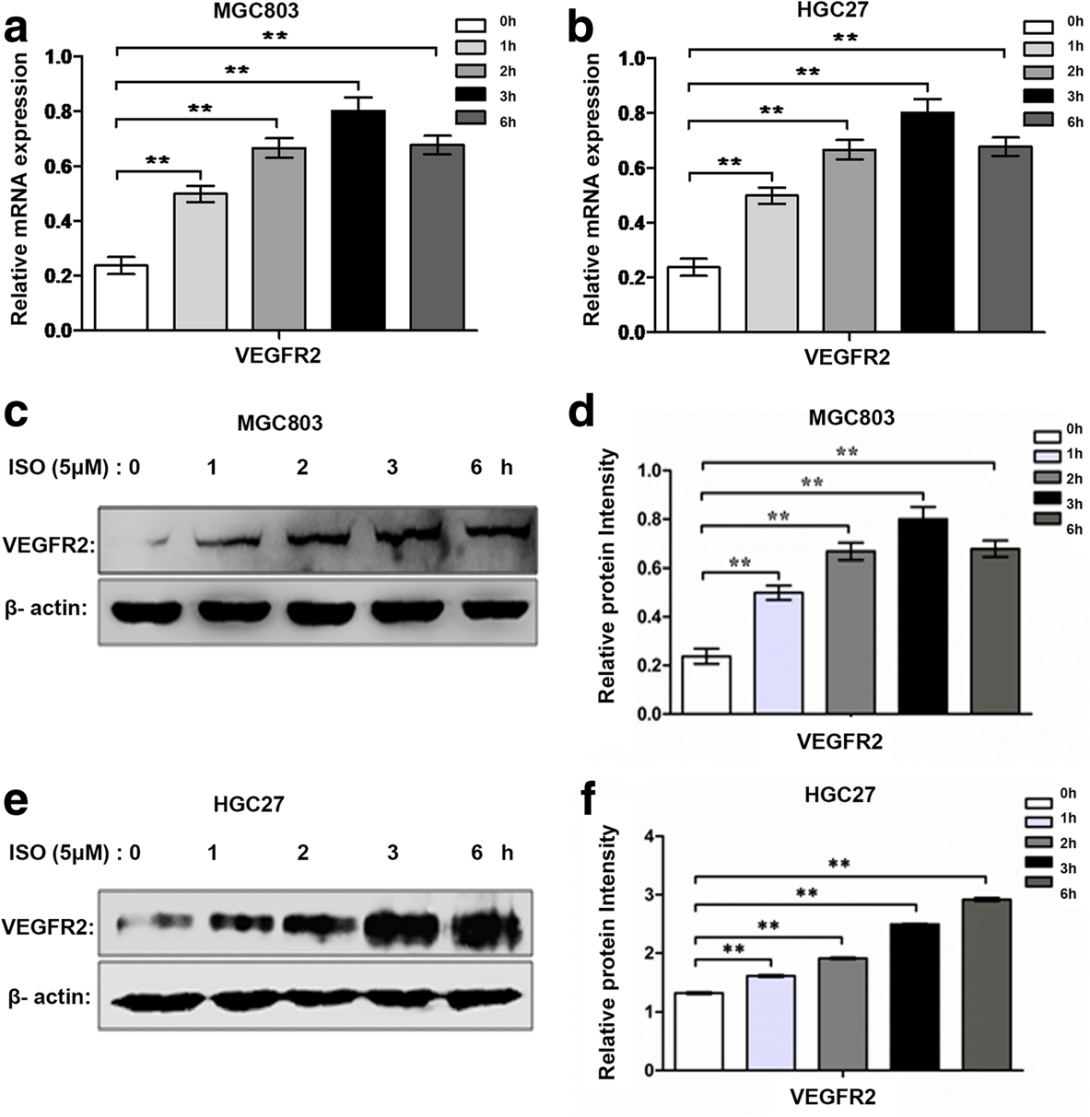
Isoprenaline promotes VEGFR2 expression in human gastric cancer cell lines. **a-f** MGC803 and HGC27 cells were treated with 5 μM ISO for 0, 1, 2, 3, and 6 h. **a-b** mRNA expression of VEGFR2 was analyzed with qRT-PCR; (**c-f**) VEGFR2 protein expression was analyzed with Western blot and quantified. Data represent means ± SD (*n* = 3 for each group, *P < 0.05, **P < 0.01)

**Fig. 8:**
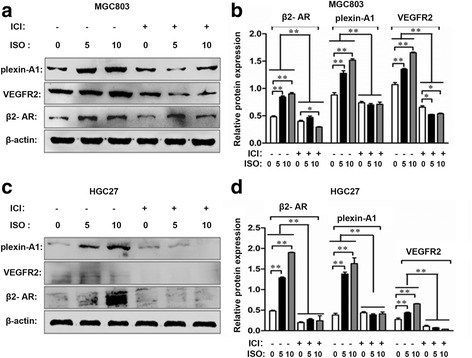
Isoprenaline promotes VEGFR2 and plexin-A1 expression via β2-AR in human gastric cancer cell lines. **a-d** MGC803 and HGC27 cells were starved overnight and incubated with 10 μM ICI 118,551 hydrochloride (ICI) (a selective antagonist of β2-AR) for 1 h to block β2-AR, in the presence of 0, 5, and 10 μM ISO for 6 h; (**a**, **c**) protein levels of plexin-A1, VEGFR2, and β2-AR were analyzed with Western blot; (**b**, **d**) the relative protein expression levels of plexin-A1, VEGFR2, and β2-AR were quantified based on the protein expression levels of β-actin. Data represent means ± SD (n = 3 for each group,*P < 0.05, **P < 0.01)

We performed real-time PCR analysis in 2 gastric cancer cell lines (HGC27 and MGC803) to determine plexin-A1 expression at transcription levels after isoprenaline treatment for 3 h. We found that isoprenaline treatment increased mRNA expression ofplexin-A1 in both MGC803 and HGC27 cell lines (Fig. 5a). Further, isoprenaline treatment increased plexin-A1 protein levels in a dose-dependent manner in both MGC803 and HGC27 cell lines (Fig. 5b-c). In addition, isoprenaline treatment increased mRNA and protein levels of plexin-A1 in MGC803 cell line in a time-dependent manner with peak levels of mRNA and protein expression at 3 h (Fig. 5d-f). Since isoprenaline acts on β2-AR, we assessed if β2-AR and VEGFR2 were co-expressed in the same cells. Indeed, we found that β2-AR and VEGFR2 were co-expressed in both tumor-associated vascular endothelial cells and gastric tumor cells, but not in normal gastric tissues (Fig. 6a-d). Then, we performed real-time PCR and western blot analysis in HGC27 and MGC803 cell lines to determine expression levels of VEGFR2 upon isoprenaline treatment. Isoprenaline treatment increased VEGFR2 expression in both cell lines at mRNA levels (Fig. 7a-b) and protein levels (Fig. 7c-f), in a time-dependent manner. To test if isoprenaline acted through β2-AR, we used ICI118, 551 (ICI, a selective inhibitor of β2-AR). We found that ICI treatment reduced β2-AR expression levels, and subsequently inhibited isoprenaline-induced up-regulation of plexin-A1 and VEGFR2 in both MGC803 cells (Fig. 8a-b) and HGC27 cells (Fig. 8c-d). Collectively, these findings indicate that isoprenaline up-regulates plexin-A1 and VEGFR2 expression via β2-AR in gastric cancer cells.

### Plexin-A1 is involved in the signals isoprenaline promoting HUVEC tube formation by induced VEGFR2

**Fig. 9:**
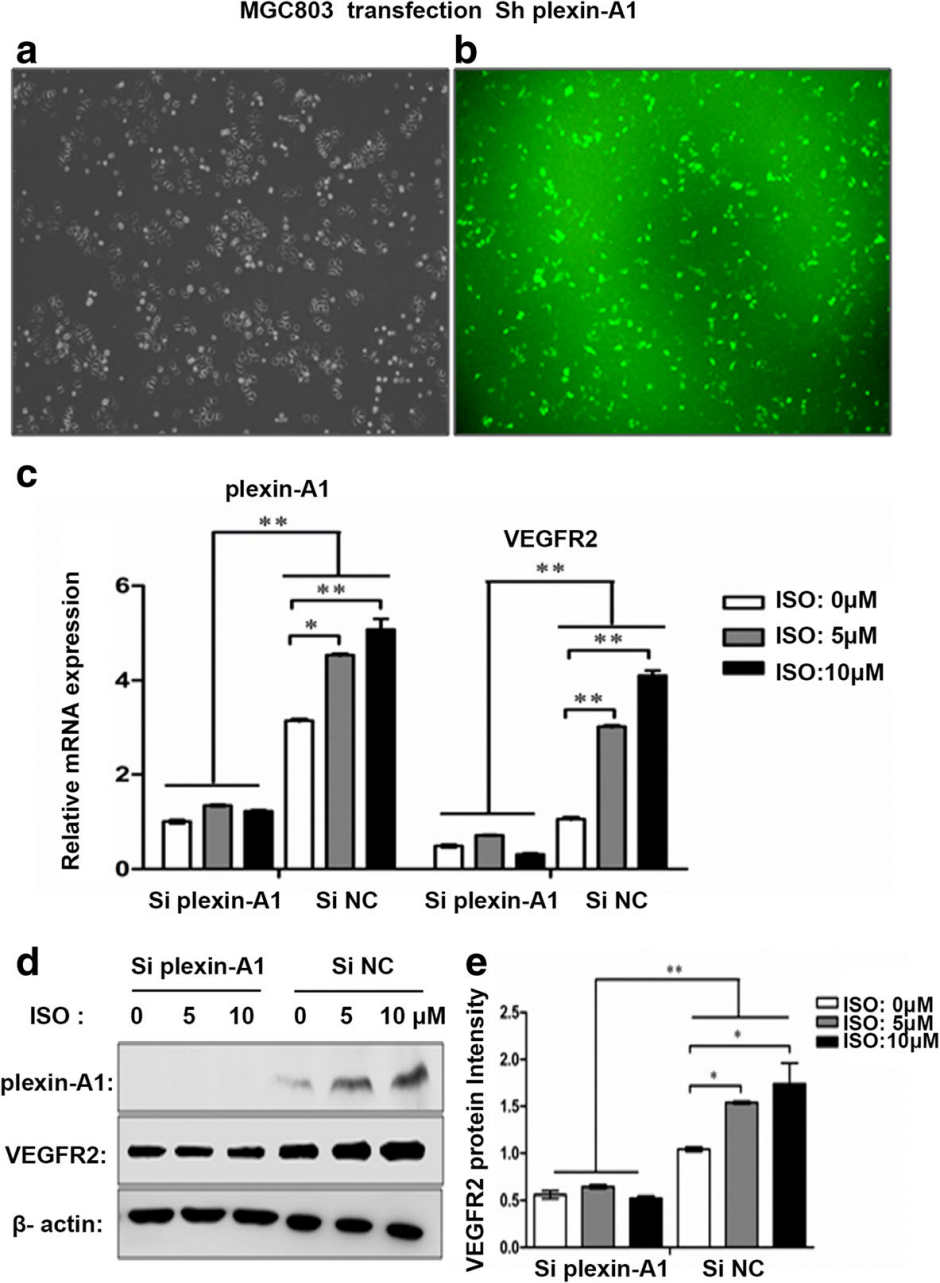
Up-regulation of VEGFR2 expression by isoprenaline is dependent on plexin-A1 expression. **a-b** MGC803 cells were transfected with shRNA/GFP, followed by G418 selection for 1 month; most cells (approximately 84%) were successfully transfected, comparing the optical microscopic image (**a**) and the fluorescence microscopic image (**b**). **c** shRNA transfected MGC803 cells were treated with 0, 5, and 10 μM ISO for 3 h, and qRT-PCR analysis of the mRNA levels of plexin-A1 and VEGFR2 in the shRNA knockdown group (Siplexin-A1) and control shRNA group (SiNC) (**d-e**) The corresponding protein levels of plexin-A1 and VEGFR2 were analyzed by Western blot and quantified. Data represent mean ± SD (n = 3 for each group, *P < 0.05, **P < 0.01)

Plexin-A1 may form a receptor complex with VEGFR2 to exert their effects on different regions of the cardiac tube [20]. This complex signals have been reported, we therefore further addressed the relationship between VEGFR2 and plexin-A1 in gastric cancer cells. We knocked down plexin-A1 expression in MGC803 cells using shRNA targeting plexin-A1. The transfection efficiency was approximately 84% based on the percentage of GFP-positive cells after transfection and antibiotics selection (Fig. 9a-b). Real-time PCR analysis indicated that knockdown of plexin-A1 decreased the basal levels of plexin-A1 and VEGFR2 expression and inhibited isoprenaline-induced up-regulation of both plexin-A1 and VEGFR2 at mRNA levels (Fig. 9c). Further, when plexin-A1 was knocked down, isoprenaline treatment was unable to up-regulate plexin-A1 and VEGFR2 expression at protein levels (Fig. 9d-e). These findings suggest that up-regulation of VEGFR2 expression by isoprenaline is dependent on plexin-A1 expression in gastric cancer cells.

**Fig. 10:**
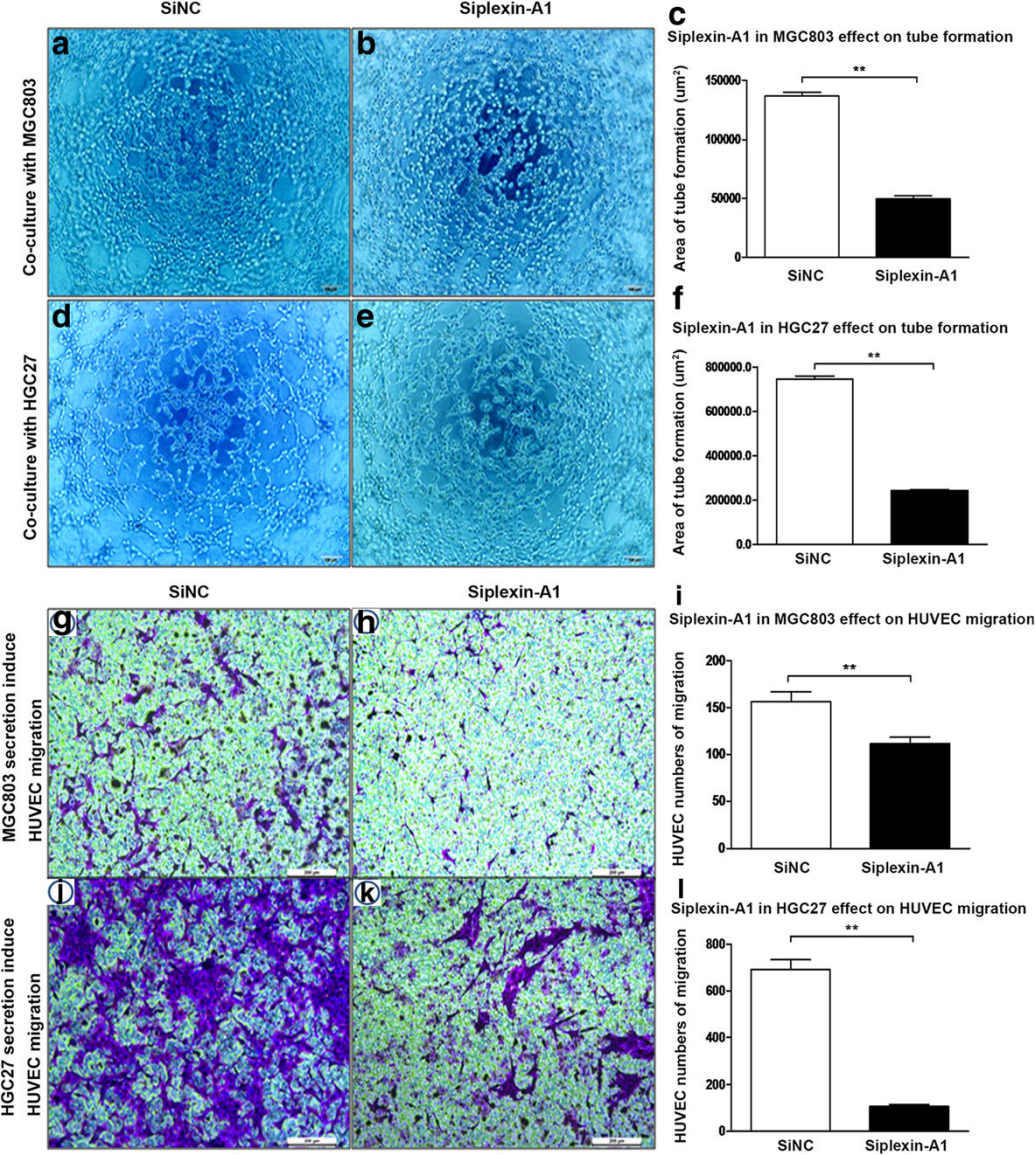
Inhibit plexin-A1 expression in tumor cells can influence HUVECs tube formation and migration. **a-f** MGC803 or HGC27 cells were divided into the shRNA knockdown group (Siplexin-A1) and control shRNA group (SiNC). HUVEC were co-culture with MGC803 (**a-b**) or HGC27 cells (**d-e**) in the matrigel coated 96-well plate. After 8 h, the tubular structures were photographed. Representative photographs (Scale bar, 100 μm) and quantitative analysis of tube area are shown in each panel (**c**, **f**). **g-l**: MGC803 (**g-h**) or HGC27 (**j-k**) gastric cancer cells (1 ml/well) were seeded bottom chambers at a concentration of 4 × 10^5^ cells/ml., which were divided into the shRNA knockdown group (Siplexin-A1) and control shRNA group (SiNC). HUVECs were suspended and planted in upper chambers. After 24 h, the migrated HUVEC were photographed (Scale bar, 100 μm) and quantitative analysis are shown in each panel (**i**, **l**). Data represent mean ± SD (n = 3 for each group,*P < 0.05, **P < 0.01)

Angiogenesis requires epithelial cells efficient migration and tube formation, thus we used transwell assay and tube formation methods to evaluate the influence of gastric cancer cells secretion on HUVEC by interfering plexin-A1 expression. In co-culture assay, we found siplexin-A1 in MGC803 (Fig. 10a-c) and HGC27 (Fig. 10d-f) can significantly decrease HUVEC tube formation. We further use the gastric cancer cells culture supernatants to detect their effect on HUVEC migration. It was shown that inhibit plexin-A1 expression in MGC803 (Fig. 10g-i) and HGC27 (Fig. 10j-l) significantly inhibit HUVEC migration. Collectively, these findings indicated plexin-A1 is involved in the signals isoprenaline acting on angiogenesis by forming the receptor complex with VEGFR2.

### Plexin-A1 konckdown reduce tumor growth in vivo

**Fig. 11:**
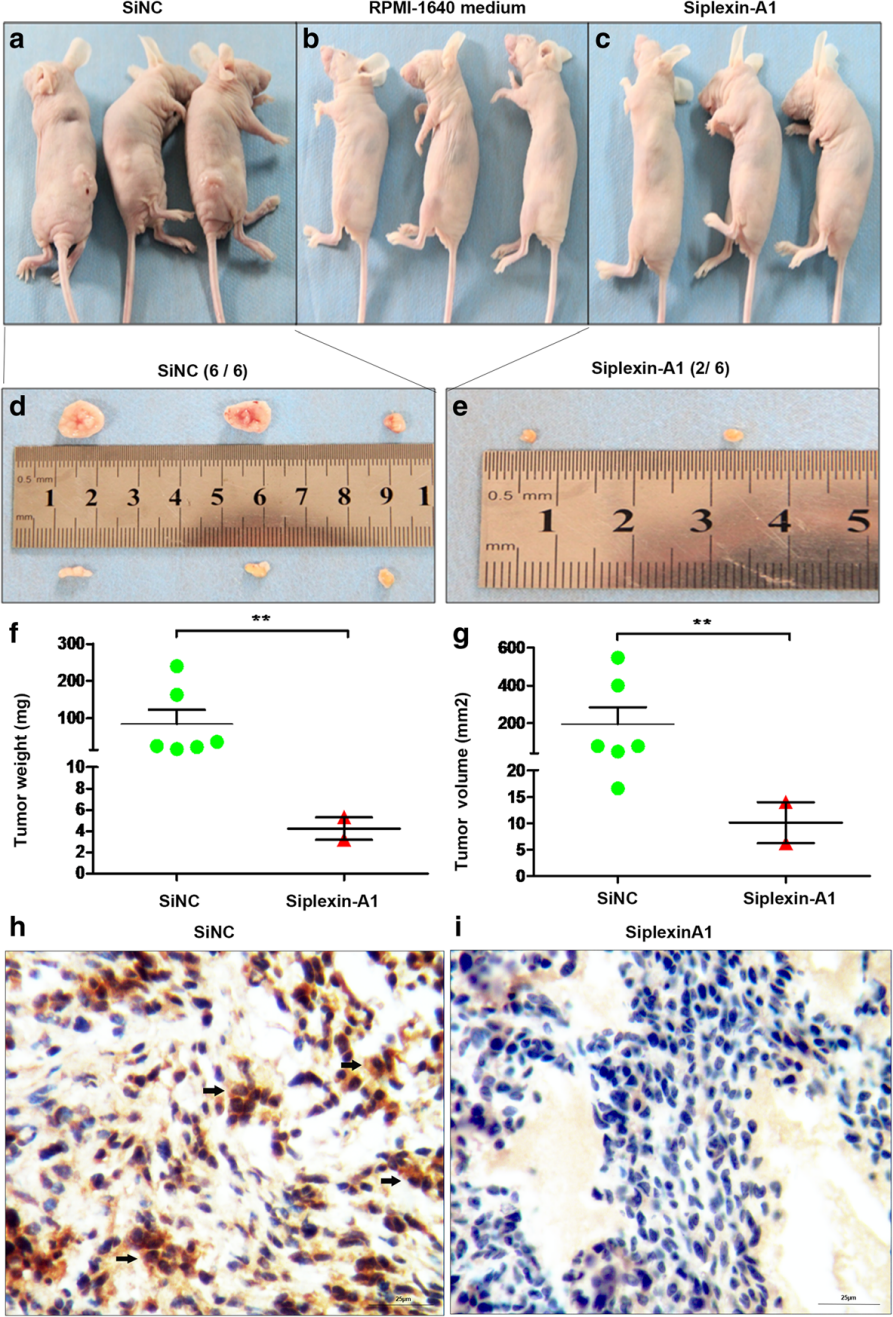
Plexin-A1 konckdown reduce tumor growth in vivo. **a-c** Representative picture of BALB/c nude mice receiving subcutaneous inoculation of shNC MGC803 cells (**a**), shPlexin-A1 MGC803 cells (**c**) or RPMI-1640medium (**b**) before excising for tumor xenografts. Representative tumor xenografts of shNC MGC803 cells (**d**) and shPlexin-A1 MGC803 cells (**e**) excised from mice were shown. There were 6/6 sites (**d**) tumor formation in siNC group, and 2/6 sites (**e**) tumor formation in siplexin-A1 group. Weight (**f**) and Volume (**g**) of tumor xenografts excised from BALB/c nude mice after plexin-A1 knockdown when compared with xenografts transfected with siNC. **h-i** Representative images of immunohistochemical staining with DAB show that plexin-A1 (black arrow) highly expressed in siNC group in xenografts (**h**), but not expressed in siplexin-A1 group (**i**). Scale bar, 25 μm

To examine whether plexin-A1 konckdown affects tumor formation in vivo, we subcutaneously inoculated shNC MGC803 cells (Fig. 11a), shPlexin-A1 MGC803 cells (Fig. 11c) or RPMI-1640 medium (blank control group, Fig. 11b) into BALB/c nude mice. In shNC group, the six sites all form xenografts (Fig. 11d). However, there are only two sixths sites form xenografts in shPlexin-A1 group (Fig. 11e). Inhibition of plexin-A1 significantly decreased the weight (Fig. 11f) and volume (Fig. 11g) of the xenografts when compared with xenografts transfected with shNC. We further systematically detected plexin-A1 expression in xenografts by immunohistochemical method (Fig. 11h-i). Plexin-A1 was highly expressed within gastric cancer cell in siNC group (Fig. 11h), but no expression in siplexin-A1 group (Fig. 11i).

## Discussion

We found that plexin-A1 and VEGFR2 proteins were highly expressed in gastric cancer cells but not in normal gastric tissues. Further, the 2 proteins were co-localized in both the tumor vessels and tumor cells. These findings suggest that plexin-A1 and VEGFR2 may be functionally related in tumor angiogenesis, which conformed to the reports that they form a receptor complex in cardiac morphogenesis.

**Fig. 12:**
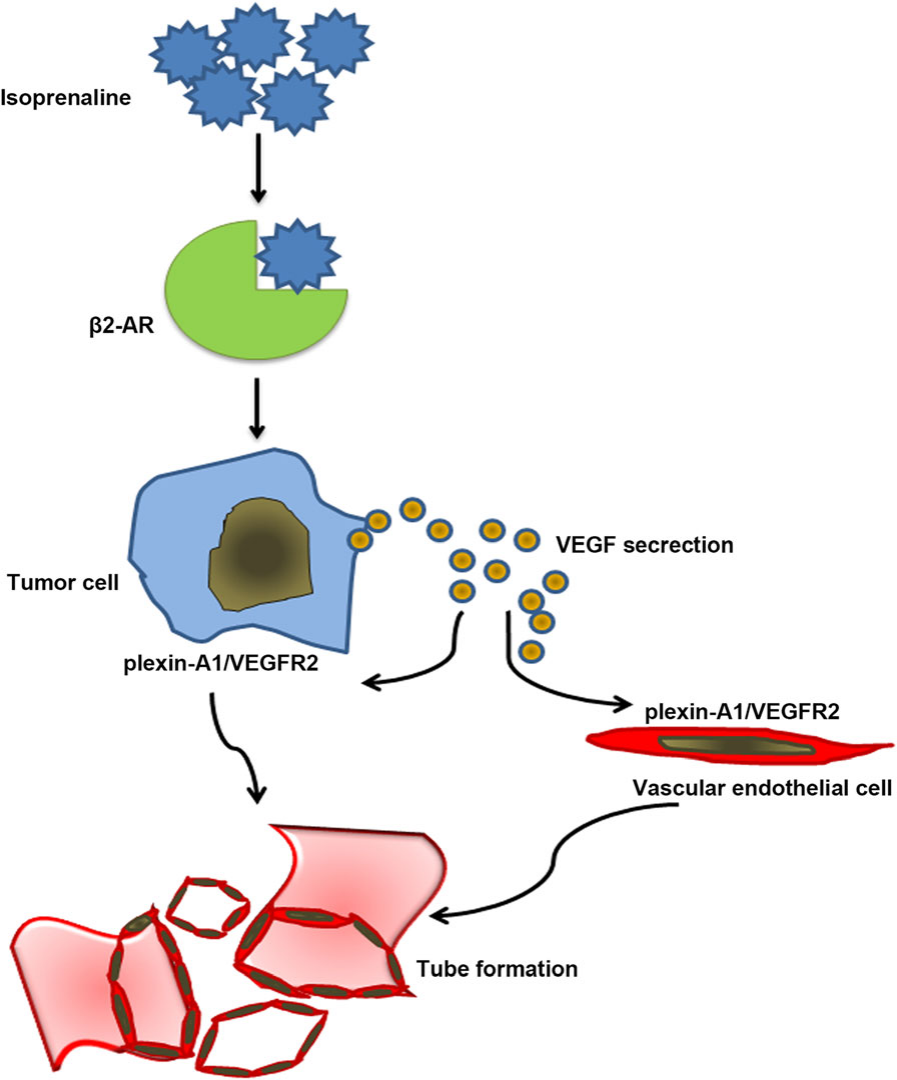
Schematic representation summarizes the results from the present study. Our studies indicated a connection between stress-related hormones and tumor angiogenesis in gastric cancer. In light of our observations, most patients with gastric cancer experienced some degree of depression, anxiety, and fear, which trigger HPA gland axis and release ISO. ISO recognized and activated their receptorsβ_2_-AR, and further stimulated VEGF secretion in gastric cancer cells, which subsequently up-regulated plexin-A1 and VEGFR2 expression in both gastric cancer cells and vascular endothelial cells. The expression of plexin-A1 in gastric cancer cells can further influence tumor angiogenesis

The biologic effects of catecholamines are mediated by α1-, α2- and β-adrenergic receptor families, which show distinct patterns of tissue distribution and signaling pathways. Among the receptors of catecholamines, the most representative one is β2-AR. Using animal models, Thaker et al. demonstrated that β2-AR was a critical mediator for stress-induced acceleration in ovarian cancer by promoting angiogenesis [16]. In another study, Lutgendorf et al. found that catecholamines significantly enhanced VEGF secretion in 2 ovarian cancer cell lines [25]. In addition, Ferrara et al. showed that the action of VEGF was mainly mediated through VEGFR2, which was present in the tumor-associated vascular endothelial cells [26]. Therefore, we were prompted to investigate whether catecholamine promote angiogenesis via VEGF secretion. Indeed, we found that β2-AR agonist isoprenaline stimulate VEGF secretion, which mainly secreted out of gastric cancer cells. Meanwhile, we found that human recombinant VEGF165 treatment up-regulate expression of plexin-A1 in both HUVECs and gastric cancer cells. Isoprenaline up-regulated expression of plexin-A1 and VEGFR2 in gastric cancer cells, and induction of VEGFR2 was dependent on expression of plexin-A1 as knockdown of plexin-A1 expression inhibited isoprenaline-induced up-regulation of VEGFR2. Subsequently, we found that knockdown of plexin-A1 expression in tumor cells effect the HUVECs tube formation, migration and tumor growth in vivo, which give us a valid support that plexin-A1/VEGFR2 signaling pathway is value in tumor angiogenesis. These findings demonstrate that isoprenaline stimulated VEGF secretion and subsequently up-regulated plexin-A1 and VEGFR2 expression in both gastric cancer cells and vascular endothelial cells. The expression of plexin-A1 in gastric cancer cells can further influence tumor angiogenesis (Fig. 12).

The present study reveals a direct link between stress-related hormones and angiogenesis in gastric cancer, which up-regulate plexin-A1 and VEGFR2 expression in gastric cancer cells to promote tumor angiogenesis via VEGF secretion. Therefore, future studies shall develop new approaches to target this signaling pathway and design new anti-angiogenic therapies for the treatment of gastric cancer.

## Conclusion

The study demonstrated the mechanism of stress contributes to tumor angiogenesis via VEGF secretion in tumor cells. It is demonstrating that VEGF promote tumor angiogenesis via plexin-A1 /VEGFR2 signaling. Plexin-A1 and VEGFR2 are co-localized in human gastric cancer cells and tumor-associated vascular endothelial cells.

## Abbreviations

DAB: 3,3′-diaminobenzidine
DAPI: 4′,6-diamidino-2-phenylindole
DAPI: 4′,6-diamidino-2-phenylindole;ICI: ICI 118,551 hydrochloride
HPA: Hypothalamic–pituitary–adrenal
HUVEC: Human umbilical vein endothelial cell
ISO: Isoprenaline
MVD: Microvessel density
qRT-PCR: quantitative real-time polymerase chain reaction
SD: Standard deviation
VEGF: Vascular epithelial growth factor
VEGFR: Vascular epithelial growth factor receptor
β2-AR: β2-adrenoceptor
